# A 2.9 GPa Strength Nano-Grained and Nano-Precipitated 304L-Type Austenitic Stainless Steel

**DOI:** 10.3390/ma13235382

**Published:** 2020-11-27

**Authors:** Congcong Du, Guoying Liu, Baoru Sun, Shengwei Xin, Tongde Shen

**Affiliations:** Clean Nano Energy Center, State Key Laboratory of Metastable Materials Science and Technology, Yanshan University, Qinhuangdao 066004, China; ccdu@stumail.ysu.edu.cn (C.D.); gyliu@stumail.ysu.edu.cn (G.L.); shengweixin@ysu.edu.cn (S.X.)

**Keywords:** nanocrystalline materials, austenitic steels, mechanical alloying, grain boundary strengthening, dispersion strengthening

## Abstract

Austenitic stainless steel has high potential as nuclear and engineering materials, but it is often coarse grained and has relatively low yield strength, typically 200–400 MPa. We prepared a bulk nanocrystalline lanthanum-doped 304L austenitic stainless steel alloy by a novel technique that combines mechanical alloying and high-pressure sintering. The achieved alloy has an average grain size of 30 ± 12 nm and contains a high density (~10^24^ m^−3^) of lanthanum-enriched nanoprecipitates with an average particle size of approx. 4 nm, leading to strong grain boundary strengthening and dispersion strengthening effects, respectively. The yield strength of nano-grained and nano-precipitated stainless steel reaches 2.9 GPa, which well exceeds that of ultrafine-grained (100–1000 nm) and nano-grained (<100 nm) stainless steels prepared by other techniques developed in recent decades. The strategy to combine nano-grain strengthening and nanoprecipitation strengthening should be generally applicable to developing other ultra-strong metallic alloys.

## 1. Introduction

Austenitic stainless steels (ASSs) have been widely used in automotive, medical equipment, and nuclear industry areas because of their well-known advantages such as excellent corrosion resistance, good plasticity, high creep resistance, and low cost. Additionally, welded structures made of austenitic steels [1,2] have also been widely used as heat exchangers, pipelines, tanks, and structural elements of ships and offshore structures. As a result, ASSs account for about 70% of the world’s stainless steel production. However, ASSs are often coarse-grained and exhibit a relatively low yield strength of 200–400 MPa [3,4]. Increasing the strength of ASSs not only has significant economic and environmental impact but also widens their applications. Thus, many techniques [5,6,7,8,9,10,11,12,13,14,15,16,17,18,19,20,21,22,23,24,25,26,27,28,29,30,31,32,33,34,35,36], as summarized in Table 1, have been utilized in the past three decades to make ultrafine-grained and nanocrystalline (NC) SSs with enhanced strength. Techniques such as cold rolling [9,14,16,17,19,20,24,34], cold rolling and annealing [5,6,7,8,11,12,13,21,27], equal channel angular processing [10,15,18,30,35], cyclic channel die compression [22], mechanical alloying and high-pressure sintering [23], high-pressure torsion [26,29], surface mechanical attrition treatment [28,32], hydrostatic extrusion [31], ultrasonic strain engineering technology [33], and multiple hot rolling [36] have been utilized to refine the grain size of SSs. As a result, the yield strength of SSs can be increased to ~1.0–2.5 GPa, which is around five to ten times that of coarse-grained ASSs. The highest yield strength, 2.5 GPa, is achieved by mechanically alloying 304L SS powders with 1 at% La in a SPEX 8000 D shaker mill and subsequently consolidating at a high pressure of 4 GPa [23]. The resultant bulk NC 304L ASS is composed of nano-sized grains (with an average grain size of 45 ± 24 nm) and (La, O, Si)-rich nanoprecipitates (with an average particle size of 4.6 ± 2.4 nm and a number density of (5.24 ± 0.25) × 10 ^23^ m^−3^) [23]. These nanoprecipitates can kinetically prevent the nano-sized grains from growing. In addition, La is also found to be segregated at the grain boundaries of matrix grains. This will lower the specific grain boundary energy, which in turn lowers the thermodynamic driving force for grain growth [23]. The combined kinetic and thermodynamic stabilization effects make it possible to achieve bulk NC 304L ASS by consolidating the mechanically alloyed NC 304L ASS powders at a relatively high temperature of 1000 °C (75% melting temperature). However, the 2.5 GPa yield strength has been simply explained by the well-known grain boundary (GB) strengthening effect [23]. Besides the above-mentioned strengthening caused by refined grains, it has also been found recently that [37] the yield strength of a coarse-grained maraging steel can be increased from 1.1 to 1.9 GPa by an extremely high density (more than 10^24^ m^−3^) of coherent nanoprecipitates. It is not unreasonable to assume that this strategy should also be applicable to SSs. In this paper, we report the microstructure and mechanical property of bulk NC lanthanum-doped 304L SS with an average grain size of 30 ± 12 nm, a high density (~10^24^ m^−3^) of coherent nanoprecipitates, and ultrahigh yield strength of 2.9 GPa. We propose that the combined effect of nano-grains and nanoprecipitates makes the lanthanum-doped NC 304L SS ultra-strong.

## 2. Materials and Methods

For the synthesis of NC lanthanum-doped alloy powders, 304L SS powder (99.9% pure, −100 mesh) and elemental metal La powder (99.9% pure, −325 mesh) supplied by Alfa Aesar were utilized. The weight ratio of 304 L SS to La was 97.5:2.5. A 200 g mixture of 304 L SS and La powders, along with 500 g hardened steel balls, were sealed into a hardened steel vial inside an argon-filled glove box (Etelux, Beijing, China) containing less than 0.1 ppm O_2_ and H_2_O. The powder mixture was then mechanically alloyed in a MiQi QM-QX2L planetary mill (Changsha Miqi Instrument Equipment Co., Ltd., Changsha, China) operated at a revolution speed of 400 r/min for 70 h. For simplicity, the resultant alloy was named 304 L-La. The as-milled NC 304 L-La powders were sintered using a CS-IB type cubic-anvil press (Guilin Metallurgical Machinery Factory, Guilin, China) under a pressure of 4 GPa at 1000 °C for 30 min. X-ray diffraction (XRD) was performed on a D/MAX-2500/PC diffractometer (Rigaku, Tokyo, Japan) with Cu Kα radiation. Transmission electron microscopy (TEM) and high-resolution TEM (HRTEM) images were acquired using a Cs-corrected Titan ETEM-G2 (FEI, Eindhoven, The Netherlands) operated at 300 KV. High-angle annular dark-field scanning transmission electron microscopy (HAADF-STEM) observation and element mapping were carried out using Cs-corrected Titan Themis Z (FEI, Eindhoven, The Netherlands) operated at 300 KV. Specimens for the TEM and HRTEM observation were prepared using a focused ion beam (FEI, Eindhoven, The Netherlands) with the ion beam operated at a voltage ranging from 2 to 30 kV. Specimens for the HAADF-STEM observation were prepared by conventional electropolishing procedures, whereby a 3 mm diameter disk was electropolished in an electrolytic solution containing 15 vol% perchloric acid and 85 vol% alcohol under a voltage of 20.5 V. A quasi-static compressive test of the sintered NC 304L-La specimens at a strain rate of 5 × 10^−4^ s^−1^ was performed using an Instron 5982 load frame (Instron, Norwood, MA, USA) equipped with a 100 kN load cell at room temperature. The specimens for compression were cylinders of 6 mm in height and 3 mm in diameter.

## 3. Results and Discussion

**Figure 1:**
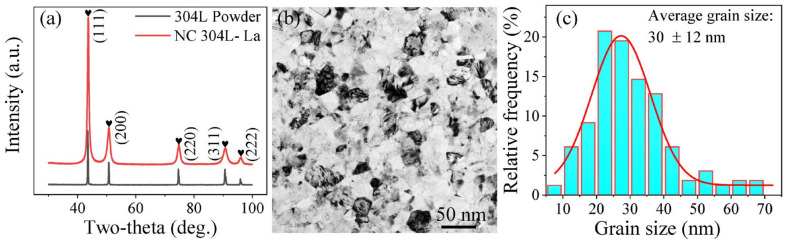
(**a**) XRD patterns of as-received 304L powder and sintered nanocrystalline (NC) 304L-La, (**b**) TEM image, and (**c**) grain size distribution of sintered bulk NC 304L-La.

Figure 1a shows the XRD patterns of as-received 304L powder and sintered bulk NC 304L-La, both of which are composed of a face-centered cubic austenitic phase, as indexed by the well-known five planes, (111), (200), (220), (311), and (222) for a face-centered cubic material. Neither body-centered cubic (or tetragonal) *α*′-martensite nor hexagonal close-packed *ε*-martensite is found—within the detection limit of XRD—in sintered bulk 304L-La. This is probably because the sintering is performed at a high temperature of 1000 °C, which can convert a martensitic phase, if any, to an austenitic phase at approx. 700 °C. The diffraction peaks of NC 304 L-La are significantly wider than those of the as-received 304 L powder, indicative of refined grains in NC 304 L-La. The TEM image of NC 304 L-La, as shown in Figure 1b, exhibits equiaxed nano-grains. The size of nano-grains takes the form of a Gaussian distribution, as shown in Figure 1c. Most nano-grains have a size between 10 and 45 nm. Counting approx. 200 grains gives an average grain size of 30 ± 12 nm.

**Figure 2:**
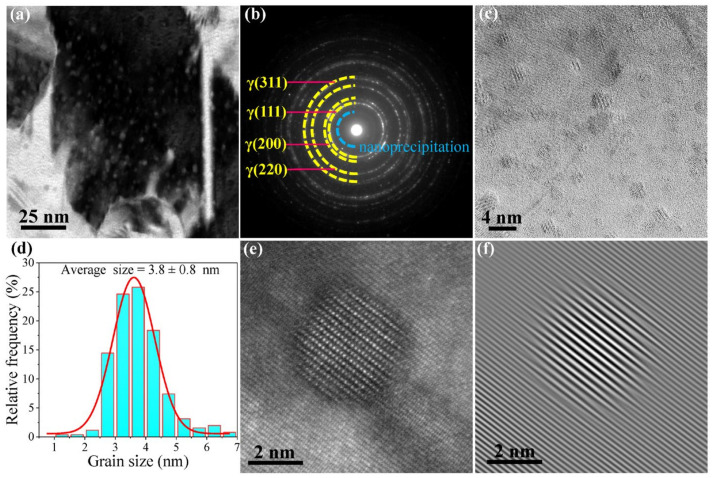
(**a**) TEM image, (**b**) selected area diffraction pattern and (**c**) high-resolution TEM (HRTEM) of NC 304L-La, (**d**) size distribution of nanoprecipitates (NPs), (**e**) high-angle annular dark-field scanning transmission electron microscopy (HAADF-STEM) image, and (**f**) the corresponding inverse fast Fourier transformation image of an NP in the grain interior.

The bright-field TEM image for NC 304L-La, as shown in Figure 2a, displays abundant nanoprecipitates (NPs) uniformly distributed in the matrix. The selected area electron diffraction patterns of NC 304L-La, as shown in Figure 2b, can be indexed as a face-centered cubic-structured matrix (yellow dotted circles) and NPs (blue dotted circle) with a lattice spacing of 0.30 nm. The HRTEM image of NC 304L-La, as shown in Figure 2c, further confirms the presence of many NPs distributed in the grain interiors and at the grain boundaries. The size of NPs, located between 1 and 7 nm and with an average value of 3.8 ± 0.8 nm, also takes the form of a Gaussian distribution, as shown in Figure 2d. The number density of these NPs is as high as 7.5 × 10^23^ m^−3^. As a result, the volume fraction *f* of these NPs reaches 2.1%. Figure 2e shows the HAADF-STEM image of an NP in the grain interior. The corresponding inverse fast Fourier transformation image, as shown in Figure 2f, indicates that the interface between the NP and the matrix is semi-coherent.

**Figure 3:**
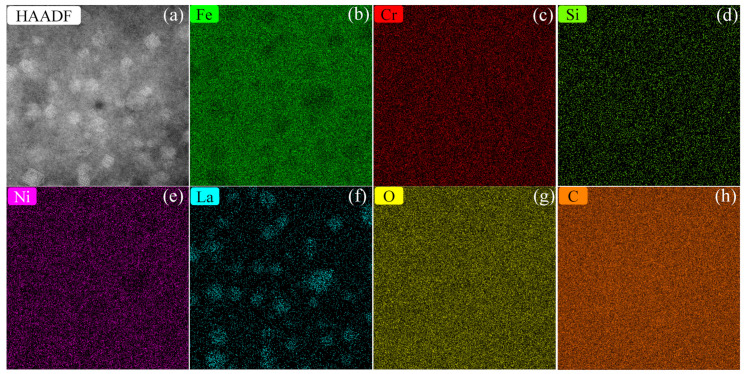
HAADF-STEM image (**a**) and the corresponding EDS elemental mapping (**b**–**h**) of NC 304 L-La.

**Figure 4:**
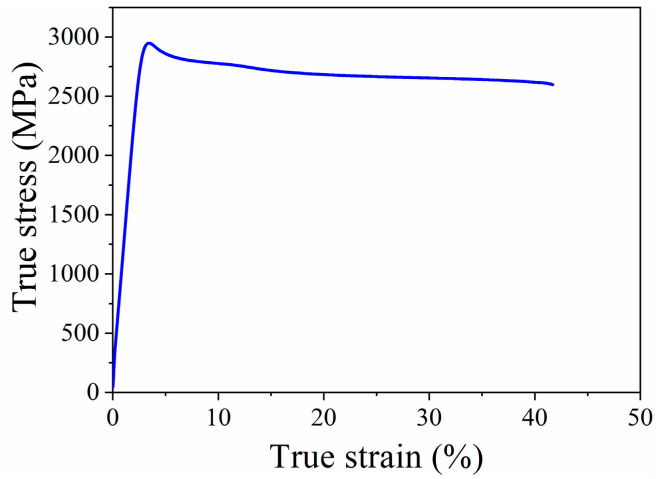
True stress–strain curve for sintered bulk NC 304 L-La under compression.

Figure 3 shows the HAADF-STEM image (a) and the corresponding energy dispersive X-ray spectroscopy (EDS) elemental mapping (b–h) of NC 304L-La. Approximately spherical NPs with a bright contrast are uniformly distributed in the matrix, as shown in Figure 3a. Figure 3b,f suggests that the NPs are depleted in Fe and enriched in La. The distribution of Cr, Si, Ni, O, and C elements, as shown in Figure 3c–e,g,h, respectively, is relatively uniform. Previously reported characterization by atom probe tomography [23] indicates that the NPs are depleted in Fe and enriched in La, O, and Si. However, the inhomogeneous distribution of relatively light elements, such as O and Si, may not be detected by the present elemental mapping technique. The fact that the NPs are depleted in Fe and enriched in La can be understood by two factors: (i) The Gibbs free energy for the formation of La oxides is more negative than that of Fe oxides [38], i.e., forming La oxides is more thermodynamically favorable than forming Fe oxides. (ii) Segregation of solute La atoms to the NP/matrix interfaces can occur because, at equilibrium, the homogeneity of the chemical potential leads to the adsorption of solute atoms on interfaces [39]. Figure 4 shows the compressive stress–strain curve of NC 304L-La, which exhibits an ultrahigh yield strength of 2909 ± 17 MPa and a large fracture strain of ~0.4. This yield strength well exceeds that (~1000–2200 MPa) of ultrafine-grained and NC SSs prepared by other techniques summarized in Table 1. A large fracture strain is indicative of the high capability for plastic deformation under compression.

Based on the above microstructural characterizations, dispersion strengthening from NPs ($\sigma_{d}$), GB strengthening ($\sigma_{gb}$) from nano-grains, and matrix strengthening ($\sigma_{m}$) should contribute to the yield strength of NC 304L-La. $\sigma_{d}$ can be expressed as [40]

(1) $$\sigma_{d}\;=\;\frac{0.8M\alpha \left(r\right)Gb}{\lambda }$$

where *M* is the mean orientation factor which is determined to be 3.06 for the face-centered cubic polycrystalline materials [41], *α*(*r*) is the dislocation obstacle strength factor, *G* is the shear modulus (73 GPa) [42], *λ* is the slip plane obstacle spacing, and *b* is the Burgers vector, which can be calculated by

(2) $$b\;=\;\frac{\sqrt{2}}{2}a$$

where *a* is lattice parameters (3.598 Å) determined by the Nelson-Riley method [43]. *λ* can be estimated from the relation:

(3) $$\lambda \;=\;2\sqrt{\frac{2}{3}}r\left[{\left(\frac{\pi }{4f}\right)}^{\frac{1}{2}}-1\right]$$

where *r* (1.9 ± 0.4 nm) and *f* (2.1%) are the average radius of NPs and the volume fraction of NPs, respectively. Equation (3) gives a λ value of 15.9 ± 3.3 nm. Odette et al. [44] have found that there is a linear correlation between *α*(*r*) and log(*r*/2*b*) when *r* > 2*b*:

(4) $$\alpha \left(r\right)\;\approx \;0.27log\left(\frac{r}{2b}\right)$$

when *α*(*r*) is in the range of 0.05 and 0.3, the particles can be sheared. *α*(*r*) is calculated as 0.154 ± 0.025 for NC 304 L-La. This means that the particles are mostly sheared, rather than dislocations bowing out between them. As a result, $\sigma_{d}$ is calculated to be 440 ± 163 MPa for NC 304 L-La.

The GB strengthening is often described by the classic Hall–Petch equation:

(5) $$\sigma_{gb}\;=\;K_{HP}D^{-1/2}$$

where *K_HP_* is the Hall–Petch coefficient and *D* is grain size. Shakhova et al. [24] have summarized the relation between the yield strength and the grain size for different Cr-Ni ASSs with a grain size between ~100 μm and 40 nm. The resultant *K_HP_* is 395 MPa·μm^1/2^, yielding a *σ_gb_* value of 2281 MPa for NC 304 L-La.

It has been widely accepted that the total strengthening can be estimated by the linear superposition method, which is based on the assumption that strengthening mechanisms operate independently [45,46]. The yield strength of NC 304L-La can then be expressed as:

(6) $$\sigma_{y}\;=\;\sigma_{m}\;+\;\sigma_{gb}\;+\;\sigma_{d}$$

where $\sigma_{d},\;\sigma_{gb},\;\mathrm{and}\;\sigma_{m}$ represent the strength contributed by dispersion strengthening from NPs, the GB strengthening from nano-grains, and the matrix strengthening, respectively. $\sigma_{m}$ has been determined to be 205 MPa for Ni-Cr ASSs [24]. The resultant *σ_y_* is 2926 ± 163 MPa, in good agreement with the experimentally measured value, 2909 ± 17 MPa.

The hardened steel balls and vial used to make NC 304L powder may introduce some iron impurities. However, the content of impurity iron is often below 1 at% [47]. Because 304L SS is mainly composed of iron, changing the content of iron in 304L SS by less than 1 at% should not significantly affect the resultant grain size and mechanical properties. However, this is not the case for the doped alloying elements. We have studied some NC 304L SSs doped by alloying elements such as Y, La, Hf, Zr, Mo, Nb, and Ta [48]. The results suggest that the grain size and the corresponding strength and plasticity of sintered 304L SSs are largely affected by the difference in the atomic radius between Fe and the doped element.

We have also consolidated the La-doped mechanically alloyed powders under a pressure of 4 GPa at a temperature between 800 and 1300 °C for 30 min. When the consolidation temperature is between 800 and 900 °C, the yield strength of as-consolidated bulk 304L ASS is between ~2700 and 2800 MPa. In addition, the as-consolidated bulk 304 L ASS is relatively brittle. Thus, a lower consolidation temperature results in insufficient sintering. When the consolidation temperature is between 1100 and 1300 °C, the grain size of as-consolidated bulk 304L ASS is between ~40 and 110 nm, leading to yield strengths of ~2780 and 2140 MPa. Thus, it should be interesting to study the processing parameter/microstructure/property relations of NC 304L SSs in future work. The processing parameters include, but are not limited to, the type and content of doped elements and the temperature, time, and pressure for sintering. Since recent results derived from molecular dynamics simulations suggest [49,50] that the plastic deformation modes and the resultant mechanical properties of NC 316L SS are largely affected by both microstructures (grain sizes) and testing conditions (strain rate and temperature), it should also be interesting to study the influence of grain size, strain rate, and temperature on the plastic deformation modes and the corresponding mechanical properties of NC 304L SS in future work.

## 4. Conclusions

In summary, a bulk NC La-doped 304 L ASS alloy with an average grain size of 30 ± 12 nm has been prepared by mechanical alloying and high-pressure sintering techniques. Abundant Fe-depleted and La-enriched NPs with an average particle size of 3.8 ± 0.8 nm and a high density of 7.5 × 10^23^ m^−3^ are observed in the bulk NC La-doped 304L ASS alloy. The NPs semi-coherently match with the matrix. The bulk NC La-doped 304L ASS alloy exhibits an ultrahigh yield strength of ~2900 MPa and a large fraction strain of ~0.4 under compression. The nano-grains and NPs provide strong GB strengthening and dispersion strengthening effects, respectively, and make the bulk NC La-doped 304L ASS alloy ultra-strong. The strategy reported here should be generally applicable to developing other ultra-strong nano-grained and nano-precipitated metallic alloys.

## Figures and Tables

**Table 1:** Phases, grain size, and yield strength of stainless steels (SSs) processed by different techniques.

Alloy	Processing Method	Phases	Grain Size (nm)	$\sigma_{0.2}\;(\mathrm{MPa})$	References
304L (La-modified)	MA + High-pressure sintering	100% γ	30 ± 12	2909 ± 17	This work
201L	95% CR at RT + 850 °C/0.5 min	14% α′ + 86% γ	65	1485	[5]
201L (Ti-modified)	90% CR at RT + 900 °C/1 min	100% γ	45	1000	[6]
301	35% CR at −10 °C + 750 °C/10 min + 60% CR at −10 °C + 850 °C/1 min	5% α′ + 95%γ	70	1970	[7]
301	95% CR at 0 °C + 850 °C/1 min	5% α′ + 95% γ	80 ± 20	1970	[8]
304	50% CR at RT	α′ + γ	-	1260	[9]
304	ECAP at 500 °C	-	50–100	1200	[10]
304	CR at −196 °C + 850 °C/4 min	28% α′ + 72% γ	300	1500	[11]
304	AR + 550 °C/2.5 min	32% α′ + 68% γ	270	1890 ± 50	[12]
304	CR at RT + 580 °C/30 min	15% α′ + 85% γ	150	1120	[13]
304	20% deformation at −196 °C	44% α′ + 6% ε+ 50% γ	-	1463 ± 16	[14]
304	ECAP at 500 °C	α′ + γ	80–100	1130	[15]
304	75% rolling at −196 °C	99% α′ + 1% γ	26 (XRD)	2054	[16]
304	40% asymmetric CR	44% α′+ 56% γ	-	1203	[17]
304L	ECAP at 700 °C	-	200–500	1121	[18]
304L	90% CR at 0 °C	98–99% α′ + 1–2% γ	-	1825	[19]
304L	CR at −153 °C to a strain of 1.8	100% α′	22 (XRD)	1590	[20]
304L	CR at RT + 600 °C/30 min	F + γ	~150	~1300	[21]
304L	Cyclic channel die compression	-	270	1023	[22]
304L (La-modified)	MA + High-pressure sintering	>95% γ	45 ± 24	2500	[23]
S304H	CR at RT to a strain of 4	65% F + 35% γ	50 ± 6	2050	[24]
316	Multidirectional forging at −196 °C	α′ + γ	36	2100	[25]
316	HPT at RT	>95% γ	40	1700	[26]
316	HPT at 400 °C	100% γ	90	1720	[26]
316L	80% CR at −196 °C + 600 °C/10 min	60% γ	100	1280	[27]
316L	SMAT at RT	100% γ	40	1450 ± 60	[28]
316L	HPT at RT + 500 °C/60 min	100% γ	53	2230 ± 50	[29]
316L	HPT at RT	100% γ	62	1360 ± 50	[29]
316L	30% CR at 250 °C + 95% CR at 15 °C + 750 °C/5 min	5% α′ + 95% γ	40 ± 10	1254	[8]
316L	ECAP at RT	-	-	1021	[30]
316L	Hydrostatic extrusion	α′ + ε + γ	40	1260	[31]
316L	SMAT at RT	α′ + γ	29	1906	[32]
316L	USET at RT	α′ + γ	10	2100	[33]
316LN	90% Cr at −196 °C	100% α′	30–50	1468	[34]
16%Cr-9%Ni ASS	ECAP at 400 °C + rolling at 400 °C	-	110	1700	[35]
18%Cr-8%Ni ASS	Multiple rolling at 500 °C	-	420 ± 60	1030 ± 75	[36]

$\sigma_{0.2}$: yield strength; ASS: austenitic stainless steel; MA: mechanical alloying; CR: cold rolling; RT: room temperature; ECAP: equal channel angular processing; AR: accumulative rolling; HPT: high-pressure torsion; SMAT: surface mechanical attrition treatment; USET: ultrasonic strain engineering technology; γ: austenite; α’: α’ martensite; ε: ε martensite; F: ferrite; XRD: X-ray diffraction.
